# Effects of a blend of green tea and curcuma extract supplementation on lipopolysaccharide-induced inflammation in horses and ponies

**DOI:** 10.7717/peerj.8053

**Published:** 2019-11-12

**Authors:** Janine Starzonek, Katja Roscher, Matthias Blüher, Dominique Blaue, Carola Schedlbauer, Manuela Hirz, Jens Raila, Ingrid Vervuert

**Affiliations:** 1 Institute of Animal Nutrition, Nutrition Diseases and Dietetics, Leipzig University, Leipzig, Saxony, Germany; 2 Equine Clinic, Internal Medicine, Justus-Liebig-University Giessen, Giessen, Hesse, Germany; 3 Division of Endocrinology and Nephrology, Department of Medicine, Leipzig University, Leipzig, Saxony, Germany; 4 Institute of Veterinary Pathology, Justus-Liebig-University Giessen, Giessen, Hesse, Germany; 5 Institute of Nutritional Science, University of Potsdam, Nuthetal Bergholz-Rehbrücke, Brandenburg, Germany

**Keywords:** Curcumin, Catechin, Equines, ER-stress, Polyphenols

## Abstract

**Background:**

In horses and ponies numerous medical conditions are known to be linked with inflammation in different tissues, especially in the liver. Besides affecting other metabolic pathways such as the expression of certain interleukins (IL), inflammation is associated with stress of the endoplasmic reticulum (ER). In particular, ER stress leads to adaptive stress response and can be measured by several markers of inflammatory and stress signalling pathways, like nuclear factor κB (NF-kB).

**Objectives:**

To investigate lipopolysaccharide (LPS)-induced inflammatory reactions and their modulation in horses and ponies by feeding a polyphenol-rich supplement consisting of green tea and curcuma.

**Methods:**

In a cross-over study, 11 animals were allocated to either a placebo or a supplement group and supplemented with 10 g of a blend of green tea and curcuma extract (GCE) or a placebo (calcium carbonate) once daily. After 21 days of supplementation, all animals underwent a LPS challenge to induce moderate systemic inflammation. Blood samples and liver biopsies were taken at standardized time points: 24 hours before and 12 hours after LPS challenge. Inflammatory blood parameters such as serum amyloid A (SAA), haptoglobin and retinol binding protein 4 (RBP4) were measured in serum. Hepatic mRNA levels of selected markers of inflammation such as *haptoglobin, tumor necrosis factor *α* (TNF-*α*), IL-1*β*, IL-6, cluster of differentiation 68 (CD68), fibroblast growth factor 21 (FGF-21), NF-*κ*B, activating transcription factor 4 (ATF4)* were quantified by RT-qPCR. In addition, liver biopsies were examined histologically for inflammatory alterations.

**Results:**

Blood markers of acute inflammatory response increased after LPS challenge. In the liver, the proinflammatory cytokine *IL-1β* showed significantly lower mRNA levels after LPS challenge in the supplemented group (*P* = 0.04) compared to the placebo group. Levels of the hepatic *CD68* mRNA increased significantly in the placebo group (*P* = 0.04). There were no significant differences between supplemented and placebo groups concerning other markers of inflammation and markers of ER stress within the liver. The number of hepatic macrophages were not different after LPS challenge in both feeding groups.

**Conclusion:**

LPS was able to induce inflammation but seemed less suitable to induce ER stress in the horses and ponies. The polyphenol-rich supplement showed some potential to reduce inflammatory responses. Nevertheless, the supplementation did not exert an overall anti-inflammatory effect in horses and ponies.

## Introduction

Horses may suffer from medical conditions that result in a low-grade inflammatory state in different tissues, especially in the liver. For example, obesity was recently suggested to induce a low-grade inflammation in horses (Vick et al., 2007). As inflammation may trigger stress of the endoplasmic reticulum (ER), equine obesity might be associated with hepatic ER stress as well (Marycz et al., 2018). ER stress is defined as an imbalance between folded and unfolded or misfolded proteins, which in consequence accumulate in the ER lumen (Zhang & Kaufman, 2008). The accumulation leads to the activation of an adaptive stress response, called the “unfolded protein response” (UPR; Kozutsumi et al., 1988; Hotamisligil, 2010). The UPR is mainly exerted by key mediators, such as nuclear factor κB (NF-κB) and results in an elevated proinflammatory response (Hotamisligil, 2010). Inflammatory cytokines not only trigger the cascade of ER stress but their genes are also known as NF-κB target genes. Therefore, after activation of transcription factor NF-κB the expression of several target genes, such as those for tumor necrosis factor α (TNF-α), interleukin 6 (IL-6), interleukin 1β (*IL-1β*), haptoglobin and serum amyloid A (SAA) are induced (Gessner et al., 2013; Chaudhari et al., 2014). Oxidative stress, an imbalance between the excessive formation of oxidants and inadequate antioxidant defenses, activates the NF-κB pathway and is therefore also directly linked with inflammation. Numerous nutraceuticals are available with claims for altering inflammatory responses and reducing ER stress. Recently, the anti-inflammatory and antioxidant properties of selected polyphenol-rich plant metabolites such as tea catechins (Gaur & Agnihotri, 2014) and curcumin, a polyphenol of *Curcuma longa* (Dulbecco & Savarino, 2013), have been the focus of interest in studies of humans and animal species such as cattle (Winkler et al., 2015) and pigs (Gessner et al., 2013). By interfering with the key regulator NF-κB (Gessner et al., 2013) the production of several inflammatory cytokines such as TNF-α and *IL-1β* are inhibited (Jurenka, 2009; Gaur & Agnihotri, 2014). As an example, flavonoids derived from grape seeds and grape marc meal had anti-inflammatory effects on TNF-α and SAA production in the duodenal mucosa of pigs (Gessner et al., 2013). Additionally, Winkler et al. (2015) described the potential to increase performance parameters in livestock by feeding a blend of polyphenols derived from green tea and curcuma extract to dairy cows. Data on anti-inflammatory and antioxidative properties of polyphenols are lacking in the equine. Therefore, the aim of the study was to investigate selected markers of inflammation in blood and liver tissue after an inflammatory stimulus with or without supplementation of a green tea and curcuma extract (GCE). We hypothesized that feeding a polyphenol-rich diet has the potential to reduce inflammation and ER stress in horses and ponies.

## Materials & Methods

### Animals

Five adult Warmblood horses and six adult Shetland ponies owned by the Institute of Animal Nutrition, Nutrition Diseases and Dietetics, University of Leipzig were used in this study. The horses had a mean (±SD) age of 19 ± 5 years and mean (±SD) body weight (BW) of 589 ± 81 kg. Mean (±SD) age of ponies was 9 ± 3 years and mean (±SD) BW was 126 ± 8 kg. The animals were housed in groups on different paddocks with a shelter hut and had free access to water and salt at all times. The project was approved by the Ethics Committee for Animal Rights Protection of the Leipzig District Government (No TVV 34/16), in accordance with German Legislation for Animal Rights and Welfare. All animals were given a thorough physical examination prior to supplementation period to determine that they were healthy.

### Supplementation period

All animals were fed meadow grass hay ad libitum before starting the experiment. During adaptation period, all animals were fed meadow grass hay (2 kg/100 kg BW) for 21 days. After adaptation a cross-over study was performed by additionally feeding either 10 g of a blend of green tea (95%) and curcuma (5%) extract (Spicemaster^©^ CP Alpha, Kaesler Nutrition GmbH, Cuxhaven, Germany; total polyphenol content of 20%) or 10 g CaCO_3_ (Kaesler Nutrition GmbH, Cuxhaven, Germany) as placebo once daily for 21 days. The supplement or the placebo were mixed in 1 kg (horses) or 0.2 kg (ponies) of a commercial feed (Pavo Pferdenahrung GmbH, Vechta Langförden, Germany) and fed to each animal individually. Intake of the supplement or placebo was monitored.

### Wash-out period

A 3 month wash-out period was conducted between the change of feeding regime according to the cross-over design. During the wash-out period, animals were fed with meadow grass hay ad libitum and had access to water and salt at all times.

### LPS challenge

After 3 weeks of supplementation animals underwent a LPS challenge. An indwelling venous catheter (Braunüle MT, B. Braun Melsungen AG, Melsungen, Germany) was aseptically inserted into the jugular vein. 10 ng/kg BW of LPS (Escherichia coli O55:B5, Sigma-Aldrich Chemie GmbH, Munich, Germany) was mixed in 1,000 mL (horses) or 500 mL (ponies) 0.9% saline (B. Braun Melsungen AG, Melsungen, Germany) and infused over 30 min. Heart rate, respiratory rate, rectal temperature, sweating, appetite, general behaviour and interaction with the examiner were monitored, using a modified pain score protocol proposed by Bussières et al. (2008) before and in 30 min intervals for 3 h after LPS challenge. Each parameter was categorized from 0 (= physiologic) to 3 (= pathologic). Clinical parameters such as colour of oral mucosa, capillary refilling time, digital pulsation and defecation were also monitored in the above-mentioned intervals.

### Blood sampling

Blood samples were collected from the jugular vein 24 h before and 12 h after LPS infusion directly into 3 blood collection tubes (Monovette, Sarstedt AG & Co. KG, Nuremberg, Germany) containing K_3_-EDTA, lithium heparin or a coagulation activator. Lithium heparin and K_3_-EDTA tubes were centrifuged immediately at 865 g for 10 min. The serum tubes were centrifuged after 30 min of clotting at room temperature under the same conditions. Serum and plasma were frozen in multiple aliquots of 1 mL at −20 °C followed by storage at −80 °C until analysis.

### Liver sampling

Liver biopsies were taken 24 h before and 12 h after LPS infusion. The animals were sedated with xylazine i.v. (0.5 mg/kg BW, Proxylaz, bela-pharm GmbH & Co. KG, Vechta, Germany). Biopsies for liver tissue sampling were performed transcutaneously on the right body side in the 12th or 13th intercostal space at the level of the tuber ischiadicum in the horses and approximately 5 cm ventrally to the level of the tuber coxae in the ponies. After clipping, surgical scrubbing, skin disinfection and injection of 2 mL lidocaine hydrochloride s.c and i.m. for local anaesthesia (20 mg/mL, Lidocainhydrochlorid 2%, bela-pharm, Vechta, Germany) a 1 cm vertical skin incision was made in the intercostal space at the cranial side of the rib. Ultrasound-guided biopsy was performed with a fully automated biopsy gun (BIP High Speed Core Cut 2, BIP Biomed. Instrumente & Produkte GmbH, Tuerkenfeld, Germany) using a 12 G x 15 cm biopsy needle. Liver samples were immediately transferred to a sterile tube (CryoPure^©^ Tube, Sarstedt AG & Co. KG, Nuremberg, Germany) and shock frozen in liquid nitrogen (−196 °C). Liver samples were then stored at −80 °C until further analysis. A second liver sample was transferred to a tube containing formalin (10%) and stored at room temperature pending histological examination.

### Analyses

#### SAA and haptoglobin

SAA (Eiken SAA, Eiken Chemical CO, Tokyo, Japan) and haptoglobin (PHASE™ RANGE Haptoglobin, Tridelta Development Limited, Maynoooth, Ireland) were measured by ABX Pentra C400 (HORIBA Europe, Darmstadt, Germany). Quality controls before measurements were performed according to the manufacturer’s instructions.

#### Retinol binding protein 4

Serum RBP4 was analysed by 12% sodium dodecyl–polyacrylamide gel electrophoresis (SDS-PAGE), using the buffer system of Laemmli (1970). After SDS-PAGE, the proteins were transferred from the gel onto a polyvinylidene difluoride membrane (Merck KGaA, Darmstadt, Germany) for 60 min, and blocked with 5% milk powder in tris-buffered saline with 0.1% Tween 20 (TBST, pH 7.6) for 1 h. The membranes were incubated for 90 min with 1:300 diluted cross-reacting rabbit anti-human RBP4 (DakoCytomation GmbH, Hamburg, Germany). After washing with 0.3% TBST, the membranes were incubated with 1:500 diluted peroxidase-conjugated sheep anti-rabbit IgG (EnVision K4003; DakoCytomation GmbH Germany) for 1 h. The colour reaction was developed using Luminol reaction (Chemiluminescence Blotting Substrate, Roche Diagnostics GmbH, Mannheim, Germany) according to the manufacturer’s instructions. Band intensity of RBP was read with an imager (Bio-Rad, Munich, Germany) and analysed with the Bio-Rad Discovery software 1.1.

### Hepatic mRNA levels

The quantity of mRNA transcripts of inflammatory cytokines in liver tissue was analysed using quantitative real-time PCR. RNA was isolated from liver tissue using a commercial kit (RNeasy^®^ Lipid tissue Mini Kit, QIAGEN GmbH, Hilden, Germany) following the manufacturer’s protocol and RNA quantity was measured by spectrophotometry (NanoVue^®^ Plus, Healthcare Biosciences AB, Munich, Germany). The RNA samples were transcribed into cDNA using a mastermix (random primer, deoxynucleotide triphosphates (dNTPs^®^), 5x First Strand Buffer, dithiothreitol (DTT)) and SuperScript™ II Reverse Transcriptase (QIAGEN GmbH, Hilden, Germany). All samples were run through standardised protocols using Peltier Thermal Cycler-200 (MJ research, St. Bruno, Canada). Gene sequences encoding the genes of interest *TNF-*α *, IL-6, IL-1*β*, NF-*κ*B, haptoglobin, CD68, FGF* 21 and *ATF4* were obtained from the database ensemble (http://www.ensembl.org). The *18S* RNA and *ribosomal protein L32 (RPL32)* were chosen as reference genes. An RNA-probe was used for the *18S* RNA. Primer (biomers.net GmbH, Ulm, Germany) validation followed standard procedures. The length varied between 16–24 base pairs, with minimum content of guanine and cytosine bases of 40–60%. When possible, more than 3 terminal t bases and more than 4 repeating structures were avoided. The program Primer3 was used to validate melting temperatures and for preventing possible secondary structures such as hairpins, homodimers and heterodimers. Table 1 gives an overview of the used primer sequences. Standard curves were generated with serial dilutions of pooled cDNA of all samples for quantification of the transcripts. For performing the quantitative real-time PCR the assays were run through a standard program of TaqMan™ (7500 Real Time PCR System, Thermo Fisher Scientific Inc., Schwerte, Germany) with minor modifications. Power SYBR Green PCR Master Mix and TaqMan™ Universal Master Mix II (Thermo Fisher Scientific Inc., Schwerte, Germany) were used. The genes of interest were normalised against the geometric mean of the two reference genes.

**Table 1:** Primer sequences used to analyse the mRNA levels of genes of interest and reference genes.

	Forward (5’-3’)	Reverse (3’-5’)
IL-6	CCACCTCAAATGGACCACTACTC	TTTTCAGGGCAGAGATTTTGC
TNF-α	AAAGGACATCATGAGCACTGAAAG	GGGCCCCCTGCCTTCT
CD68	CTTTGGGCCAAGTTTCTCTTGT	AAGAGGCCGAGGAGGATCAG
IL-1β	CGGCAATGAGAATGACCTGT	GCTTCTCCACAGCCACAATG
Haptoglobin	AGAAAGCAGCCTGTGGAGAT	AGCCAGACACATAACCCACA
NF-kB	GCTTTGTGACAAGGTGCAGA	ACGATCATCTGTGTCTGGCA
FGF-21	GATGATGCCCAGGAGACAGA	AAGTGGAGCGATCCGTACAG
ATF4	TGGTCTCAGACAACAGCAAG	AGCTCATCTGGCATGGTTTC
RPL32	AGCCATCTACTCGGCGTCA	TCCAATGCCTCTGGGTTTC

### Immunohistochemistry

Immunohistochemistry was performed on liver biopsies using an antibody against lysozyme (rabbit pAb, A099, Dako Deutschland GmbH, Hamburg, Germany). Sections were deparaffinized in xylene substitute (Roti-Histol, Carl Roth GmbH & Co KG, Karlsruhe, Germany) and rehydrated using a descending alcohol series. Blocking of endogenous peroxidase activity was carried out by incubation in methanol with 0.5% hydrogen peroxide. Slides were pretreated with 0.05% protease for antigen retrieval. The primary antibody was diluted 1:600 in tris-buffered saline (TBS) and was incubated at 4 °C overnight. As a secondary antibody pig anti rabbit IgG (Vector Laboratories, Burlingame, California) was used together with rabbit PAP (peroxidase-anti-peroxidase; Z113, Dako Deutschland GmbH, Hamburg, Germany) as detection system. Antibody binding was visualized using 3,3′-diaminobenzidine (DAB, Sigma-Aldrich, St. Louis, MO) as chromogen, and sections were counterstained with Papanicolaou solution (Merck KGaA, Darmstadt, Germany). Slides were dehydrated in an ascending alcohol series and mounted with a xylene-based solution. All liver biopsies were examined and semi-quantitatively evaluated dependant on the number of macrophages within each liver biopsy as follows: grade 0 = no macrophages, grade 1 = mild (1–33 macrophages), grade 2 = moderate (34–66 macrophages) and grade 3 = severe (67–100 macrophages). For each sample the number of macrophages was counted in 5 high power fields (HPF, 400x total magnification). The examiner was blind to the treatment group of origin of the samples.

### Statistics

The commercial statistical software program STATISTICA^©^  (StatSoft GmbH, Hamburg, Germany) was used for statistical analyses. All data on blood and liver parameters were checked for normal distribution by using Shapiro–Wilks test. As data set was not normally distributed Wilcoxon test for non-parametric data was used for analyses. Level of significance was set at *P* < 0.05. Data are shown as median and 25./75. percentiles. Immunohistochemical results were assessed and are presented descriptively.

## Results

There were no feed refusals of the compound feed mixed either with GCE or placebo for horses and ponies. In addition, there were no recorded hay refusals in either group during the supplementation period.

### LPS challenge

All animals showed behavioural signs of discomfort (e.g., yawning, pawing and anorexia) up to 180 min after LPS infusion (data not shown). Rectal temperature increased significantly (*P* < 0.0001) from mean (±SD) basal temperature of 37.3 ± 0.31 °C up to mean (±SD) maximum temperature of 38.5 ± 0.59 °C after LPS challenge.

### Blood parameters

#### SAA and haptoglobin

**Figure 1:**
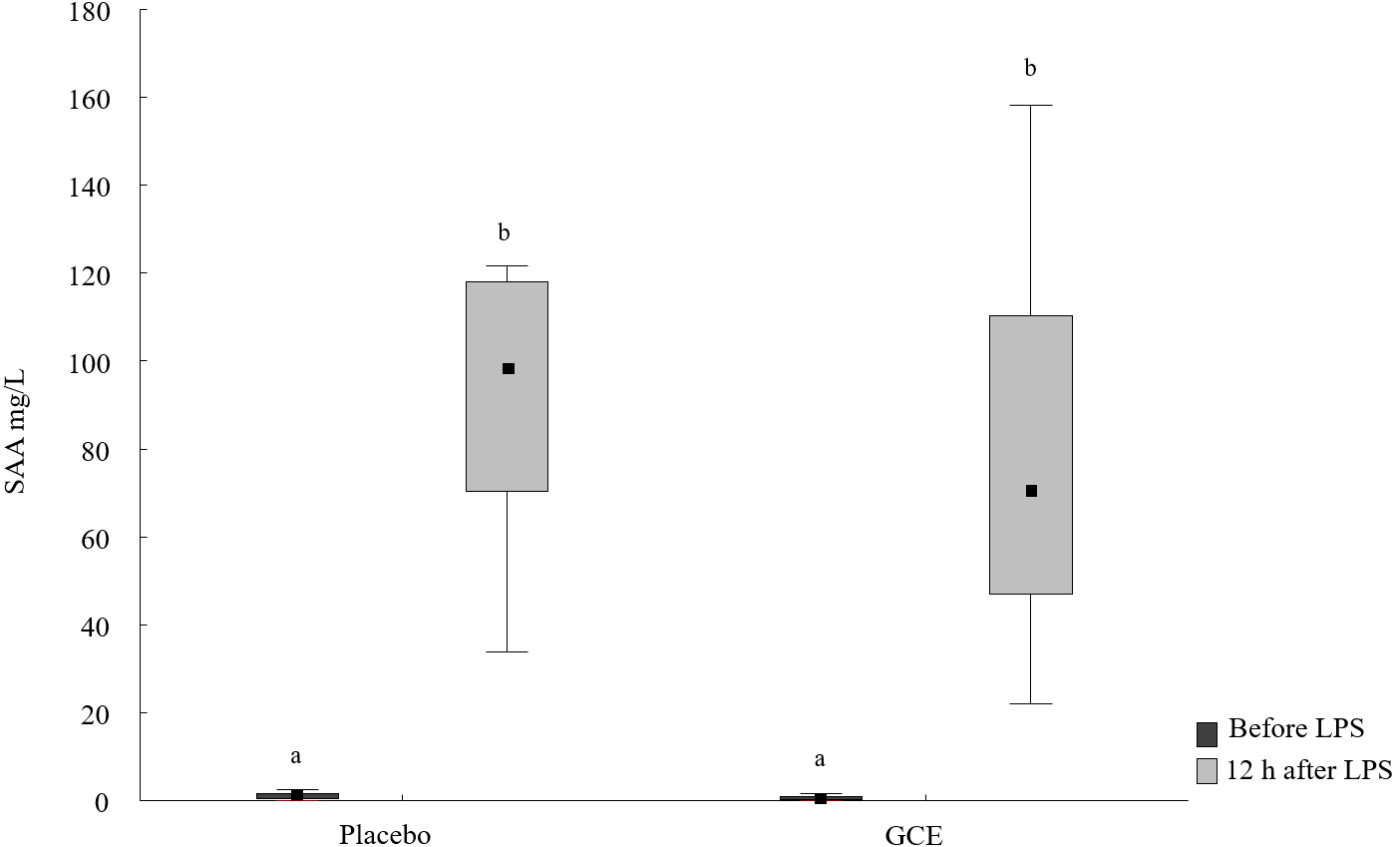
SAA concentrations before and 12 hours after lipopolysaccharide (LPS) challenge in horses and ponies fed placebo or green tea and curcuma extract (GCE). Data are shown as median (squares), 25./75. percentiles (boxes), minimum and maximum (whiskers). Different superscript letters indicate significant differences (*P* < 0.05).

SAA concentrations increased 12 h after LPS challenge compared to basal SAA levels for both the GCE group (*P* = 0.003) and placebo group (*P* = 0.008, see Fig. 1 and Table 2). The increase in SAA was not significantly different between GCE and placebo supplementation groups. Twelve hours after LPS infusion, haptoglobin increased compared to baseline samples in the GCE supplemented animals (*P* = 0.005; see Table 2) but not in the placebo group. However, serum haptoglobin levels were not significantly different between feeding groups at both time points.

**Table 2:** Markers of inflammation in serum before and 12 hours after lipopolysaccharide (LPS) challenge in horses and ponies fed placebo or green tea and curcuma extract (GCE). Values are presented as median (25./75. percentiles). Different superscript letters indicate significant differences (*P* < 0.05) within a row.

Parameter	Placebo		GCE	
	Before LPS	12 h after LPS	Before LPS	12 h after LPS
SAA [µg/mL]	1.2^a^ (0.3/1.9)	98.4^b^ (70.2/118)	0.6^a^ (0.1/1.2)	70.7^b^ (46.8/111)
Haptoglobin [mg/mL]	1.5^ab^ (1.1/1.8)	1.6^ab^ (1.2/2.5)	1.3^a^ (1.2/1.7)	1.7^b^ (1.3/1.8)
RBP4 [µg/mL]	4.7^a^ (3.5/5.9)	4.2^a^ (2.7/5.6)	4.2^a^ (3/6.1)	4.1^a^ (2.5/5.6)

#### Serum RBP4 concentrations

Serum RBP4 concentrations did not show significant changes, either by LPS stimulus or between feeding group (see Table 2).

### Hepatic mRNA levels

**Figure 2:**
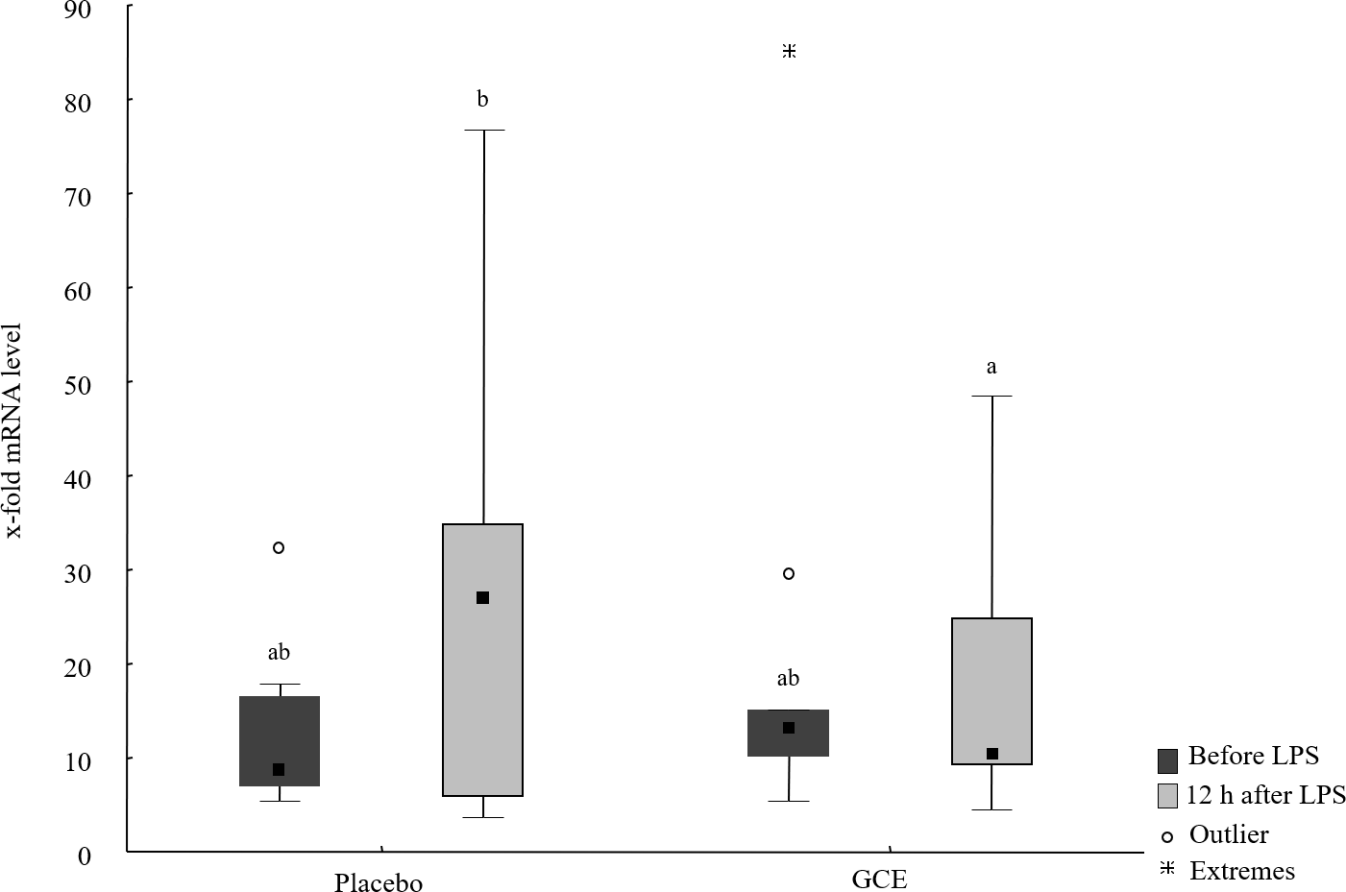
Liver mRNA levels of IL-1β before and 12 hours after lipopolysaccharide (LPS) challenge in horses and ponies fed placebo or green tea and curcuma extract (GCE). Data are shown as median (squares), 25./75. percentiles (boxes), minimum and maximum (whiskers). Different superscript letters indicate significant differences (*P* < 0.05).

Baseline mRNA levels of all hepatic parameters were not different between the GCE group compared to placebo group. mRNA levels of *TNF-*α*, IL-6, NF-*κ*B* and *ATF4* did not show significant changes induced by LPS or by supplementation. In the placebo group, mRNA levels of *haptoglobin* were higher (*P* = 0.03) after LPS challenge compared to baseline. In GCE group, LPS challenge did not significantly change mRNA levels of *haptoglobin*. However, mRNA levels of *haptoglobin* were not significantly different between feeding groups at either time point. Levels of *CD68* mRNA were higher (*P* = 0.04) after LPS challenge compared to baseline in the placebo group but not in the GCE group. Comparing both feeding groups 12 h after LPS revealed no significant differences in mRNA levels of *CD68*. After LPS challenge mRNA levels of *IL-1*β** tended to be higher in placebo group but differences were not statistically significant. Levels of *IL-1*β** mRNA in GCE group were not altered significantly by LPS challenge. Twelve hours after LPS, GCE group had significantly lower mRNA levels in *IL-1*β** than did the placebo group (*P* = 0.04, see Fig. 2). Levels of *FGF21* mRNA did not show significant changes induced by LPS challenge in both feeding groups although levels in GCE group showed a trend towards reduction after LPS challenge (*P* = 0.05, see Table 3). Levels of *FGF21* mRNA were not significantly different between feeding groups after LPS.

**Table 3:** Hepatic mRNA levels of markers of inflammation and stress of the endoplasmic reticulum (ER) before and 12 hours after lipopolysaccharide (LPS) challenge in horses and ponies fed placebo or green tea and curcuma extract (GCE). Values are presented as median (25./75. percentiles). Different superscript letters indicate significant differences (*P* < 0.05) within a row.

Parameter	Placebo		GCE	
	Before LPS	12 h after LPS	Before LPS	12 h after LPS
TNF-α	7.66^a^ (2.98/10.3)	4.37^a^ (3.94/9.68)	4.32^a^ (3.12/6.2)	7.88^a^ (3.86/12)
Haptoglobin	1.03^a^ (0.87/1.33)	1.35^b^ (1.24/1.49)	1.18^ab^ (0.64/1.7)	1.51^ab^ (1.15/1.7)
NF-κB	2.21^a^ (1.18/3.76)	2.12^a^ (1.63/3.32)	2.04^a^ (1.59/2.87)	3.70^a^ (2.22/4.63)
ATF4	1.87^a^ (0.96/3.24)	1.38^a^ (1.06/1.58)	1.72^a^ (1.23/2.77)	1.19^a^ (0.51/1.87)
CD68	4.58^a^ (2.8/9.45)	8.63^b^ (6.02/11.8)	5.49^ab^ (3.65/6.65)	11.58^b^ (6.96/16)
FGF21	1.46^ab^ (0.12/5.18)	0.15^b^ (0.05/0.59)	1.65^a^ (0.14/6.36)	0.08^ab^ (0.02/1.6)
IL-6	7.71^a^ (3.64/74.9)	4.29^a^ (2.05/8.22)	1.81^a^ (1.7/3.78)	3.55^a^ (2.35/9.08)
IL-1β	8.63^ab^ (6.91/16.6)	26.93^b^ (5.79/34.9)	13.19^ab^ (10.13/15.1)	10.53^a^ (9.14/24.9)

### Immunohistochemistry

Macrophages were found in all liver samples (see Table 4). In the placebo group the amount of grade 1 samples was lower after LPS challenge (10%) compared to before LPS (30%). In contrast, the number of grade 2 and 3 samples was higher after LPS challenge (grade 2: 50 to 60%; grade 3: 20 to 30%). Contrary to placebo, the GCE group had a higher number of grade 1 samples from 60 to 70% after LPS challenge, whereas the number of grade 2 samples was lower (30 to 20%). The number of samples staged as grade 3 in GCE group remained unchanged after LPS challenge. Comparing feeding groups after LPS stimulus, most liver biopsies of the placebo group (60%) had a moderate number of hepatic macrophages (grade 2) whereas in the GCE group most liver biopsies (70%) had a low number of hepatic macrophages (grade 1, see Table 4).

**Table 4:** Hepatic number of macrophages before and 12 hours after lipopolysaccharide (LPS) challenge horses and ponies fed placebo or green tea and curcuma extract (GCE). Values are presented as absolute numbers and percentages of the subpopulation. Grade 0 = no macrophages, grade 1 = 1-33 macrophages, grade 2 = 34-66 macrophages, grade 3 = 67-100 macrophages.

Grade	Placebo		GCE	
	Before LPS	12 h after LPS	Before LPS	12 h after LPS
0	0/10	0/10	0/10	0/10
1	3/10 (30%)	1/10 (10%)	6/10 (60%)	7/10 (70%)
2	5/10 (50%)	6/10 (60%)	3/10 (30%)	2/10 (20%)
3	2/10 (20%)	3/10 (30%)	1/10 (10%)	1/10 (10%)

## Discussion

In the present study, we hypothesized that feeding a polyphenol-rich diet for 21 days to equines would mitigate the inflammatory response in blood and liver tissue to an inflammatory stimulus. For this purpose, we used a feed additive consisting of green tea and curcuma extract with a total polyphenol content of 20%. As reviewed by Gaur & Agnihotri (2014), green tea provides one of the best sources for polyphenols. Products based on green tea contain high amounts of catechins as the active polyphenols. Catechins such as epigallocatechin gallates belong to the group of flavonoids (Lipiński et al., 2017). The polyphenol curcumin is one of three groups of curcuminoids and the main component of turmeric, a spice which is derived from the plant *Curcuma longa* (Jurenka, 2009).

In the literature, there are equivocal results concerning inflammatory parameters response to the effect of polyphenols on different tissues and animal species and more particularly between *in vitro* and *in vivo* studies. Tea catechins and curcumin demonstrated anti-inflammatory activity *in vitro* in human epithelial cells (Jobin et al., 1999; Wheeler et al., 2004; Biswas et al., 2005). However *in vivo*, the generally poor bioavailability of oral polyphenols is a challenge in the field of nutraceuticals since Nakagawa, Okuda & Miyazawa (1997) found only 0.2–2.0% of epigallogatechin-3-gallate to be available in human plasma after oral administration. Levels of tea catechins in blood and liver tissue have also been studied previously by Kim et al. (2000) in rats and mice after repeated oral doses of green tea. The authors demonstrated that green tea catechins could be detected in several tissues in these species. Nevertheless, liver tissue seems to not be significantly affected by tea catechins, as the hepatic levels were low compared to other tissues such as intestines or bladder (Kim et al., 2000). To the best of our knowledge, there are very few studies on the bioavailability of polyphenols in equines. Wein & Wolffram (2013) found quercetin, a flavonoid, to be abundant in equine plasma which is only comparable to some of the polyphenols we used in our study. Still, no data on the availability of polyphenols derived from green tea and curcuma in equine plasma is available. A limitation of the current study was that plasma and tissue levels of polyphenols were not examined in our animals but bioavailability of polyphenols was presumed to be low due to the minor dietary effects by the GCE post LPS challenge.

Nevertheless, the selected dosage of polyphenols in the present study was in the upper range of what was found in the literature. Winkler et al. (2015) fed a product consisting of green tea and curcuma at an estimated dose of 0.04–0.1 g polyphenols per 100 kg BW to dairy cows. In the present study horses were fed 0.3 g per 100 kg BW of polyphenols daily, and ponies were given 1.6 g per 100 kg BW. However, most effects in livestock were found in performance parameters such as a higher milk yield in dairy cows (Winkler et al., 2015) or an improved gain to feed ratio in pigs (Gessner et al., 2013). Performance parameters were not in the focus in the present study as the horses and ponies were kept under maintenance conditions and were adults and non-reproductive.

Regarding the daily doses of GCE in our animals, the ponies were given approximately 5 times higher doses per 100 kg BW compared to the horses. However, there was no significant difference in hepatic *IL-1β* mRNA levels comparing ponies and horses which underlines the assumed low bioavailability of the used polyphenols.

According to recent studies in livestock, a polyphenol-rich diet might be beneficial for animals such as cattle and pigs whose demands of high growth and milk production performance can trigger chronic inflammatory conditions (Gessner et al., 2013; Winkler et al., 2015). Since it was postulated by Winkler et al. (2015) that cattle are usually kept under conditions of highly elevated metabolic stress and chronic inflammation we used a LPS challenge as a well-established inflammatory model to induce a low grade inflammation in the equine animals (Oliveira-Filho et al., 2011; Tadros & Frank, 2012; Vinther et al., 2015). In consequence, we were able to evaluate the discussed anti-inflammatory effects of GCE supplementation under standardized inflammatory conditions. In the present study, inflammation induced by LPS challenge was verified by several inflammatory parameters in blood and liver tissue such as increased SAA and serum haptoglobin concentrations and increased mRNA levels of *CD68* and *haptoglobin* in liver tissue. According to Jacobsen et al. (2006), in healthy horses SAA concentrations were found to be ≤ 2.3 mg/L by using turbidimetry as the measurement principle. Therefore, all of our animals were within or close to the postulated range with all values of SAA being ≤ 2.6 mg/L prior to LPS challenge. Additionally, all animals were in the reference range of 2–10 g/L for haptoglobin according to a review by Crisman, Scarratt & Zimmerman (2008) which included different analytical methods, such as turbidimetry. The suitability of SAA and haptoglobin as inflammatory markers in horses was demonstrated by Hultén et al. (2002) where both parameters significantly increased due to an experimentally-induced non-infectious arthritis. Therefore, the SAA response to LPS induced inflammatory conditions by increasing to more than 100-fold higher concentrations compared to baseline was expected. Contrary to our hypothesis, feeding the GCE did not have an anti-inflammatory effect on the inflammatory blood parameters since concentrations did not differ from placebo group. However, it remains open if a longer lasting sampling procedure, e.g., 24 h after LPS might have an impact on the outcome of SAA and especially on haptoglobin as both parameters seem to reach their peak values later than 12 h after an inflammatory stimulus. Although levels of SAA start to increase early at 6–12 h after inflammation, peak values can be expected after 36–48 h (Hultén et al., 2002; Jacobsen & Andersen, 2007). Serum haptoglobin increased significantly 24 h after inflammation with peak values at 48–96 h (Hultén et al., 2002; Vinther et al., 2016).

As reviewed by Gessner, Ringseis & Eder (2017), oxidative stress is directly linked with inflammation in farm animals. RBP4, which is mainly produced in the liver, seems to play a role in both conditions. Besides its carrier function as specific plasma retinol transporter (Zabetian-Targhi et al., 2015), RBP4 was suggested to have a role in the induction of oxidative stress and vascular inflammation *in vitro* in human endothelial cells through activation of NF-κB (Farjo et al., 2012). Since serum levels of RBP4 and mRNA levels of *NF-*κ*B* in the liver in the current study were not altered by the inflammatory stimulus, the comparably low dose of LPS seemed less suitable to induce oxidative stress in our horses and ponies. Other parameters that are related to oxidative stress were not examined.

Macrophage invasion from blood into affected tissues during inflammation plays a crucial role in the progression of inflammation (Imamura et al., 2005). Liver macrophages, called Kupffer cells (KC), are promotors of the inflammatory cascade in hepatic tissue. We observed changes in numbers of hepatic macrophages but with a maximum variation of only 20% in course of treatment with LPS in both feeding groups. Since the results on hepatic macrophages should only be used as numerical references, the effect of GCE feeding and LPS challenge remains open. However, due to the changes we found in the liver samples, this might be an interesting aspect of further research. Furthermore, the invasion of macrophages is, among other things, mediated by release of cytokines such as TNF-α, IL-1β and IL-6 (Tacke, 2012) which mostly failed to be induced in our horses and ponies by the LPS challenge. Besides immunohistochemistry of KCs, Baldus et al. (1998) proposed analysis of CD68 to be a sensitive method to specify the amount of KCs as an increased number of hepatic CD68^+^ macrophages were found in conditions of moderate and severe inflammation such as hepatitis B. Cai et al. (2005) identified the role of CD68 as a marker for the activation of resident KCs. As mRNA levels of *CD68* increased after LPS challenge in the placebo group, but not in the GCE group, GCE seemed to have an inhibiting effect on the activation of KCs and might therefore impair the subsequent inflammatory processes in the liver. However, a significant difference between the two feeding groups concerning macrophage activation could not be demonstrated. It can be concluded that our low-dose-LPS challenge might not have caused a significant invasion of hepatic macrophages according to the few numerical changes in liver samples. Due to elevated mRNA levels of *CD68* an increased activation of resident KCs can still be assumed.

We also investigated mRNA levels of different inflammatory markers to assess protective effects of GCE on inflammatory signalling pathways in the liver. Haptoglobin is mainly produced in liver tissue and was induced under conditions of acute inflammation in rat liver cells (Wang et al., 2001). Our findings confirmed that *haptoglobin* was induced by LPS in liver tissue of horses and ponies, since it showed a significant increase after LPS challenge in the placebo group. However, a significant increase was not observed in the GCE group. In accordance with our hypothesis, we found an anti-inflammatory effect by GCE on *IL-1β*, a proinflammatory cytokine, as hepatic mRNA levels after LPS challenge were significantly lower in the GCE group compared to the placebo group. These findings agree with *in vitro* studies on mouse fibroblasts, where polyphenols were able to reduce expression of *IL-1β* through inhibition of the NF-κB pathway (Gonzales & Orlando, 2008). In a cell culture study curcumin reduced the production of proinflammatory cytokines when treating isolated lymphocytes collected from old horses with polyphenols (Siard, McMurry & Adams, 2016). In addition, tea catechins reduced activation of NF-κB in LPS-treated mouse-derived peritoneal macrophages *in vitro* (Lin & Lin, 1997). Gessner et al. (2013) used a polyphenol-rich diet consisting of grape seed and grape marc meal extract and found *NF-kB* and other target genes, like *TNF-*α** and *SAA* to be suppressed in the duodenal mucosa, but not in the liver in pigs. Similarly, in the present study, *NF-*κ*B* and its target genes, such as *TNF-*α** and *IL-6*, were unaltered at all time points. Due to animal welfare concerns liver tissue was only obtained once after LPS challenge. Therefore, the second sampling point was set at 12 h after LPS but might have not been able to detect a presumably early reaction of the examined cytokines.

Beside the above discussed parameters of inflammation, upregulation of FGF21 has been postulated as a sensitive marker of ER stress as FGF21 levels increased in rat hepatocytes *in vitro* and *in vivo* due to experimentally induced ER stress (Schaap et al., 2013). Under conditions of ER stress FGF21 is induced via an ATF4 dependent pathway where ATF4 acts as a transcriptional effector in the UPR cascade (Schaap et al., 2013; Kim et al., 2015; Wan et al., 2018). Liver *FGF21* mRNA levels were reduced in dairy cows in the peri-parturient phase and therefore under elevated metabolic stress by feeding an extract consisting of green tea and curcuma similar to the additive used in the present study (Winkler et al., 2015). Nevertheless, to the best of the author’s knowledge, the properties of FGF21 and ATF4 in conditions of ER stress have not been previously reported in equine animals. In contrast to Winkler et al. (2015), hepatic levels of *FGF21* and *ATF4* in our study were not significantly altered by feeding GCE. Although LPS was shown to induce ER stress *in vitro* in mice (Endo et al., 2006), the LPS challenge did not induce *FGF21* and *ATF4* in our animals and was therefore apparently insufficient to induce significant hepatic ER stress, presumably due to a low dosage. As a result, the evaluation of the impact of GCE supplementation on ER stress was limited.

## Conclusion

In conclusion, our findings indicate that feeding a supplement containing green tea and curcuma extract has some potential to mediate inflammatory reactions in horses and ponies. However, an overall anti-inflammatory effect by the polyphenols was not apparent. Inflammation in our animals was indicated by several inflammatory parameters in blood and liver tissue but the parameters that are linked to ER stress remained unchanged throughout the study. This suggests that the inflammatory model was not suitable to induce ER stress in our animals, leaving the effects of flavonoids and curcuminoids on hepatic ER stress uncertain in this species. Further studies are needed for a better understanding of the bioavailability and tissue distribution of polyphenols from green tea and curcuma extract in horses and ponies.

##  Supplemental Information

10.7717/peerj.8053/supp-1 File S1 Raw data on hepatic mRNA levels and blood related parameters

10.7717/peerj.8053/supp-2 File S2 Comparison of hepatic mRNA levels of markers of inflammation and stress of the endoplasmic reticulum (ER) before and 12 hours after lipopolysaccharide (LPS) challenge in horses and ponies fed placebo or green tea and curcuma extract (GCE) Values are presented as median (25./75. percentiles).

## Additional Information and Declarations

### Data Availability

The following information was supplied regarding data availability: The raw measurements are available in the Supplemental File.
