# Supplementary material for: Effects of a blend of green tea and curcuma extract supplementation on lipopolysaccharide-induced inflammation in horses and ponies
Source: PeerJ. 2019 Nov 12;7:e8053. doi: 10.7717/peerj.8053 (PMC6857679; doi:10.7717/peerj.8053)
Supplement: File S2 — Values are presented as median (25./75. percentiles). [file peerj-07-8053-s002.docx]

**Comparison of hepatic mRNA levels of markers of inflammation and stress of the endoplasmic reticulum (ER) before and 12 hours after lipopolysaccharide (LPS) challenge in horses and ponies fed placebo or green tea and curcuma extract (GCE).**

| Parameter | Breed | Placebo | | GCE | |
| --- | --- | --- | --- | --- | --- |
|  |  | Before LPS | 12 h after LPS | Before LPS | 12 h after LPS |
| TNF-α | Horses | 4.66  (3.05/7.66) | 4.05 (3.3/5.76) | 5.09 (4.46/6.2) | 7.14 (3.17/12.28) |
|  | Ponies | 8.86 (2.98/39.04) | 5.76 (4.17/14.58) | 5.26  (3:12721.6) | 4.31 (3.54/7.88) |
| Haptoglobin | Horses | 1.09 (0.84/1.48) | 1.35 (1.05/1.37) | 0.92 (0.87/1.33) | 1.35 (1.25/1.39) |
|  | Ponies | 1.32 (0.61/1.7) | 1.68 (1.59/2.2) | 1.16 (1.01/1.3) | 1.31 (1.24/1.49) |
| NF-κB | Horses | 1.59 (1.52/2.21) | 2.12 (1.63/2.49) | 1.59 (1.14/2.39) | 1.71 (1.59/3.7) |
|  | Ponies | 3.74 (1.18/3.78) | 2.57 (1.74/4.95) | 1.92 (1.77/4.42) | 2.25 (1.46/3.88) |
| ATF4 | Horses | 1.01 (0.98/2.08) | 1.36 (1.06/1.54) | 1.71 (1.43/1.74) | 1.28 (1.19/1.62) |
|  | Ponies | 2.55 (0.96/3.47) | 1.48 (1.24/2.17) | 2.02 (1.23/2.77) | 0.89 (0.49/1.87) |
| CD68 | Horses | 4.76 (4.58/4.93) | 6.34 (6.02/9.27) | 5.01 (3.65/5.49) | 7.3 (7/8.25) |
|  | Ponies | 3.46 (2.7/9.45) | 9.77 (7.52/11.84) | 5.29 (5.01/5.51) | 7.46 (3.12/16.02) |
| FGF21 | Horses | 1.12 (1.05/1.95) | 0.15 (0.05/2.87) | 1.65 (0.39/6.36) | 0.08 (0.07/0.39) |
|  | Ponies | 2.57 (0.12/5.69) | 0.19 (0.11/0.31) | 1.69 (0.14/5.06) | 0.81 (0.01/2.74) |
| IL-6 | Horses | 7.71 (3.64/11.18) | 2.47 (1.38/2.89) | 2.67 (1.81/3.78) | 9.08 (2.35/13.22) |
|  | Ponies | 36.25 (5.75/206.85) | 7.18 (4.29/15.75) | 2.57 (1.69/31.75) | 3.39 (2.74/3.55) |
| IL-1β | Horses | 8.89 (5.97/16.56) | 26.93 (16.51/31.32) | 12.4 (10.79/14.95) | 12.86 (11.74/24.9) |
|  | Ponies | 8.09 (6.99/12.63) | 17.6 (4.5/34.88) | 13.41 (10.13/15.11) | 9.85 (9.14/10.53) |

Values are presented as median (25./75. percentiles).
